# Supplementary material for: Assessment of Lab4P Probiotic Effects on Cognition in 3xTg-AD Alzheimer’s Disease Model Mice and the SH-SY5Y Neuronal Cell Line
Source: Int J Mol Sci. 2023 Feb 28;24(5):4683. doi: 10.3390/ijms24054683 (PMC10003662; doi:10.3390/ijms24054683)
Supplement: Supplementary file 1 [file ijms-24-04683-s001.zip › ijms-2136424-supplementary.pdf]

Supplementary information:

Assessment of Lab4P Probiotic Effects on Cognition in 3xTg-AD Alzheimer's Disease Model Mice and the SH-SY5Y Neuronal Cell Line

[Webberley, T.S. *et al*]

Supplementary Table S1. Compositional breakdown of the chow diet. Data are obtained and modified from Teklad diet formulations provided by Envigo.

Primary Ingredients<br>(Order of Inclusion)	Wheat<br>Corn<br>Wheat Midds<br>Soybean meal<br>Corn gluten meal<br>Soy oil
Protein %	18.6
Fat %	6.2
Metabolizable Energy	3.1 kcal/g<br>13.0 kJ/g
Isoflavone content	150-340 mg/kg
Life stage	Breeding, growth
Purpose and Benefits	Breeding<br>Growth<br>Maintenance<br>General Purpose

Supplementary Table S2. Compositional breakdown of the HFD. Data tables obtained and modified from Special Diet Services (SDS).

		FRESH	10% H_2O
TOTAL	%	100.00	100.00
Moisture	%	7.46	10.00
Crude Oil	%	22.27	21.66
Crude Protein	%	19.87	19.32
Crude Fibre	%	3.91	3.80
Ash	%	5.24	5.10
NFE	%	41.02	39.89
Pectin	%	0.46	0.45
Hemicellulose	%	3.17	3.08
Cellulose	%	4.85	4.72
Lignin	%	0.43	0.42
Starch	%	34.74	33.79
Sugar	%	1.24	1.21
SDS Gross Energy	MJ/kg	19.67	19.13
SDS Digestible Energy	MJ/kg	17.66	17.13
SDS Metabolisable Energy	MJ/kg	16.54	16.09
AFE Energy	%	18.56	18.05
C14 1 Myristoleic	%	0.03	0.03
C16 1 Palmitoleic	%	0.11	0.11
C18 1 W9 Oleic	%	6.69	6.51
C18 2 W6 Linoleic	%	1.95	1.90
C18 3 W3 Linoleic	%	0.11	0.11
C20 4 W6 Arichidonic	%	0.03	0.03
C22 5 W3 Clupanadonic	%	0.00	0.00
C12:0 Lauric	%	0.03	0.03
C14:0 Myristic	%	0.37	0.36
C16:0 Palmitic	%	4.56	4.43
C18:0 Stearic	%	2.04	1.98
Arginine	%	0.71	0.69

Lysine	%	1.16	1.13
Methionine	%	0.48	0.47
Cystine	%	0.09	0.09
Tryptophan	%	0.18	0.18
Histidine	%	0.48	0.47
Threonine	%	0.70	0.68
Isoleucine	%	0.97	0.94
Leucine	%	1.60	1.56
Phenylalanine	%	0.85	0.83
Valine	%	1.17	1.14
Tyrosine	%	0.83	0.81
Taurine	%	0.00	0.00
Glycine	%	0.83	0.81
Aspartic Acid	%	1.09	1.06
Glutamic Acid	%	3.34	3.25
Proline	%	1.40	1.36
Serine	%	0.79	0.77
Hyd. Proline	%	0.01	0.01
Hyd. Lysine	%	0.00	0.00
Alanine	%	0.57	0.55
Ca	%	0.66	0.64
P Total	%	0.57	0.55
P Phytate	%	0.05	0.05
P Available	%	0.52	0.51
Na	%	0.23	0.22
Cl	%	0.38	0.37
K	%	0.65	0.63
Mg	%	0.17	0.17
Fe	mg/kg	107.84	104.88
Cu	mg/kg	15.32	14.90
Mn	mg/kg	61.83	60.13
Zn	mg/kg	53.72	52.25

Co	µg/kg	537.57	522.81
I	µg/kg	12.74	12.39
Se	µg/kg	45.28	44.04
F	mg/kg	4.67	4.54
Vitamin A	iu/kg	6575.48	6395.00
Vitamin D3	iu/kg	8050.00	7829.05
Vitamin E	iu/kg	56.95	55.39
Vitamin B1 Thiamine	mg/kg	6.52	6.34
Vitamin B2 Roboflavin	mg/kg	4.64	4.51
Vitamin B6 Pyridoxin	mg/kg	5.82	5.66
Vitamin B12 Cyanocobalamine	µg/kg	5.45	5.30
Vitamin C Ascorbic Acid	mg/kg	7.97	7.75
Vitamin K	mg/kg	4.91	4.78
Folic Acid	mg/kg	0.15	0.15
Nicotinic Acid	mg/kg	21.44	20.85
Pantothenic Acid	mg/kg	13.30	12.93
Choline	mg/kg	278.07	270.44
Inositol	mg/kg	908.50	883.56
Biotin	µg/kg	56.35	54.80

Supplementary Table S3: Oligonucleotide sequences. Abbreviations: *Bax*, *Bcl-2*-associated X apoptosis regulator; *Bcl-2*, B-cell lymphoma 2 gene; *Iba-1*, ionized calcium-binding adaptor protein-1; *IL*, interleukin; *TNF-α*, tumour necrosis factor-α; *SLC18A*, solute carrier family 18 member 2; *PSEN1*, Presenilin-1; *CDK5*, Cyclin-dependent kinase; *NeuN*, neuronal nuclei antigen; *TH,* tyrosine hydroxylase.

Gene	Organism	Forward primer (5′ to 3′)	Reverse primer (5′ to 3′)
Bax	Mouse	TGCAGAGGATGATTGCTGAC	GATCAGCTCGGGCACTTTAG
Bcl-2	Mouse	AGTACCTGAACCGGCATCTG	GCTGAGCAGGGTCTTCAGAG
Iba-1	Mouse	TGAGGAGATTTCAACAGAAGCTGA	CCTCAGACGCTGGTTGTCTT
IL-1β	Mouse	GAGGACATGAGCACCTTCTTT	GCCTGTAGTGCAGTTGTCTAA
IL-6	Mouse	GAGGATACCACTCCCAACAGACC	AAGTGCATCATCGTTGTTCATACA
IL-8	Mouse	CTAGGCATCTTCGTCCGTCC	TTCACCCATGGAGCATCAGG
IL-10	Mouse	TAAGGGTTACTTGGGTTGCCA	GAGAAATCGATGACAGCGCC
TNF-α	Mouse	GTCCCCAAAGGGATGAGAAGT	TGGTTTGCTACGACGTGGG
β-actin	Mouse	ACACCCGCCACCAGTTCGCCAT	CACACCCTGGTGCCTAGGGCGGCCCACGATG
SLC18A	Human	GTCCTCGGAAGAGCATCG	CACACGATAACAAGCACCAG
PSEN1	Human	GGTGAATATGGCAGAAGGAGAC	AGGGCTTCCCATTCCTCACTG
CDK5	Human	CGAGAAACTGGAAAAGATTG	TTCAGAGCCACGATCTCATG
NeuN	Human	TCGAGGACACCTTGACTTCGG	GCTGCTGTGACTTCAAGCTGGT
TH	Human	GCCCTACCAAGACCAGACGTA	CGTGAGGCATAGCTCCTGA
Bax	Human	CCCGAGAGGTCTTTTTCCGAG	CCAGCCCATGATGGTTCTGAT
Bcl-2	Human	CATGTGTGTGGAGAGCGTCAA	GCCGGTTCAGGTACTCAGTCA
IL-1β	Human	TTCGAGGCACAAGGCACAA	TGGCTGCTTCAGACACTTGAG
IL-6	Human	TCTGCCAGTGCCTCTTTGCT	TTCGGTACATCCTCGACGGC
IL-8	Human	GAAGTTTTTGAAGAGGGCTGAGA	TTTGCTTGAAGTTTCACTGGCA
IL-10	Human	GCAGAGTGAAGACTTTCTTTCAAATG	TGGCAACCCAGGTAACCCTTA
TNF-α	Human	TGCACTTTGGAGTGATCGGC	TTGTCACTCGGGGTTCGAGA
β-actin Housekeeper	Human	ACTCTTCCAGCCTTCCTTCC	CGTACAGGTCTTTGCGGATG

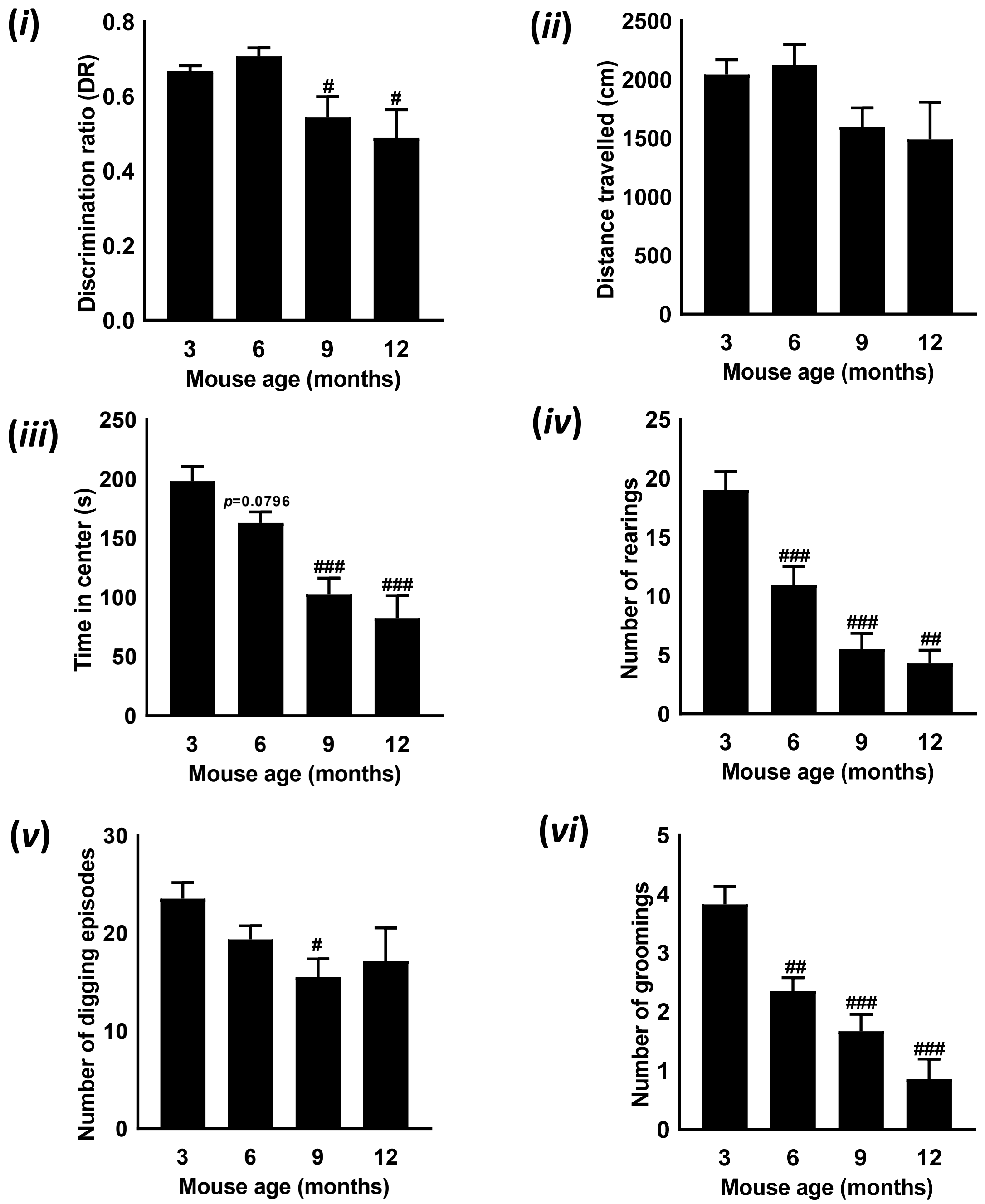

Supplementary Figure S1: Temporal changes in (*i*) discrimination ratio determined during novel object recognition testing or (*ii*) total distanced travelled, (*iii*) time in centre of arena, (*iv*) number of rearings, (*v*) number of digging episodes and (*vi*) number of groomings as determined during open field testing of 3xTg-AD mice receiving chow diet for 12 months. Data are expressed as mean ± standard error of the mean (SEM). Mouse numbers for each data point were: 54 mice (3 and 6 months), 18 mice (9 months) and 8 mice (12 months). Values of p were determined using one-way ANOVA with Tukey's host hoc analysis or the Kruskal-Wallis test with Dunn's *post hoc* where $^{\#}p<0.05$, $^{\#\#}p<0.01$ or $^{\#\#\#}p<0.001$ or as stated versus the 3 month measurements.

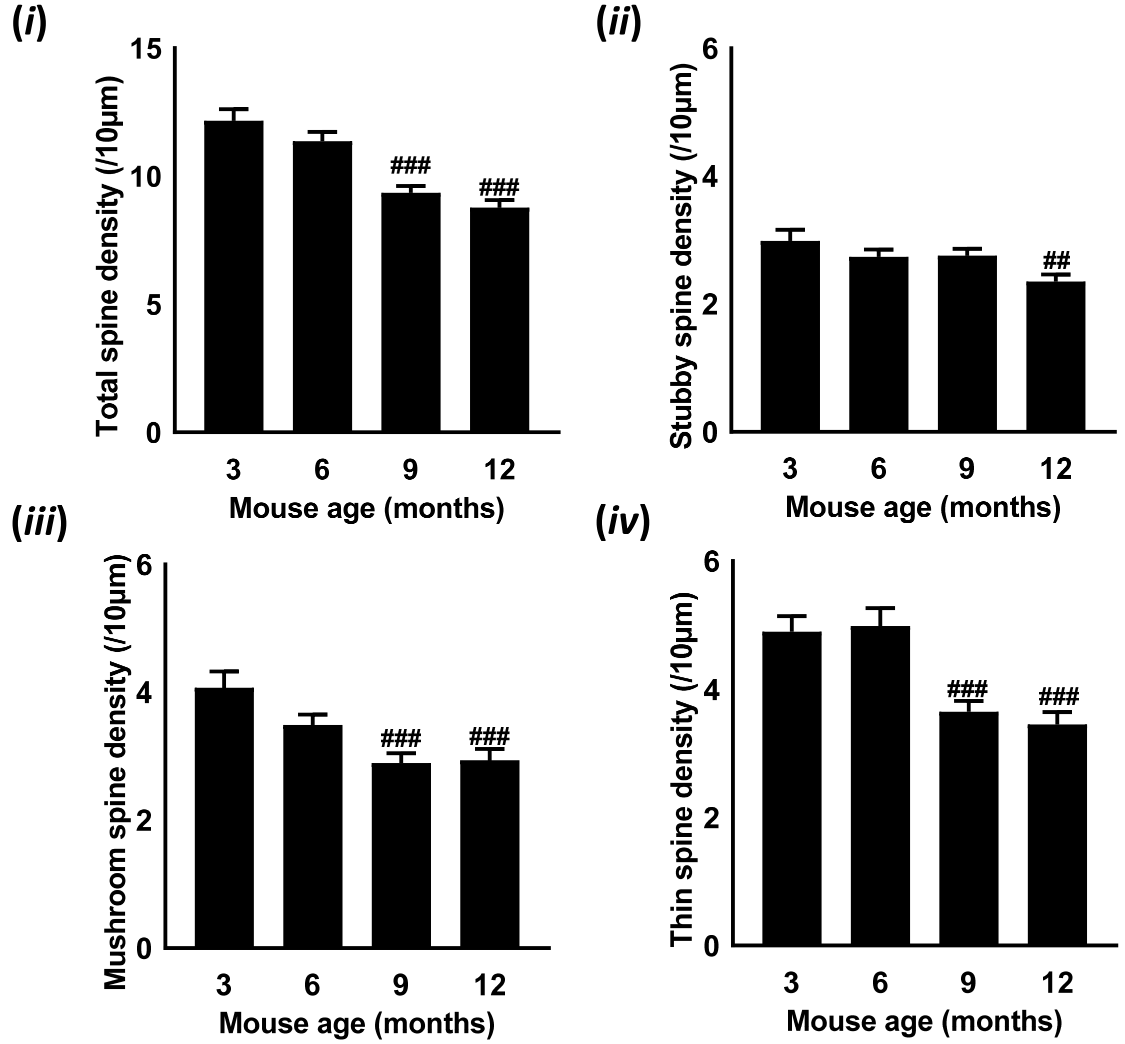

Supplementary Figure S2: Temporal changes in (*i*) total spine density, (*ii*) stubby spine density, (*iii*) mushroom spine density and (*iv*) thin spine density in the hippocampus of 3xTg-AD mice receiving chow diet for 12 months. Data are expressed as mean ± standard error of the mean (SEM). Mouse numbers were 5 per timepoint (3, 6, 9 and 12 months). Values of *p* were determined using one-way ANOVA with Tukey's host hoc analysis where $^{\#\#}p<0.01$ or $^{\#\#\#}p<0.001$ versus the 3 month measurements.

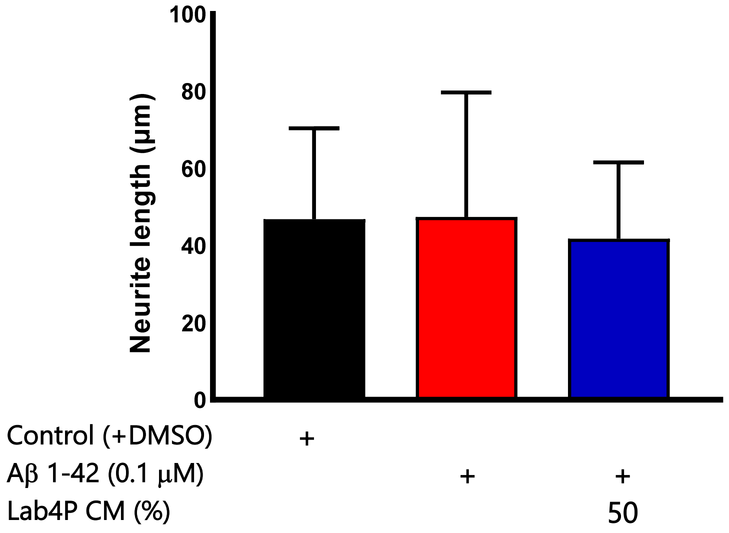

Supplementary Figure S3: Neurite length of RA/BDNF differentiated SH-SY5Y cells that were untreated (vehicle control (DMSO)) or treated with 0.1 M β-Amyloid 1-42 for 48 hours with or without 24 hours preincubation with 50% Lab4P CM. The data are presented as the mean ± standard error of the mean (SEM) of 3 independent experiments (with triplicate samples). Values of *p* were determined using the Kruskal-Wallis test with Dunn's *post hoc*. Abbreviations: RA, retinoic acid; BDNF, brain derived neurotrophic factor; DMSO, dimethyl sulphoxide; CM, conditioned media

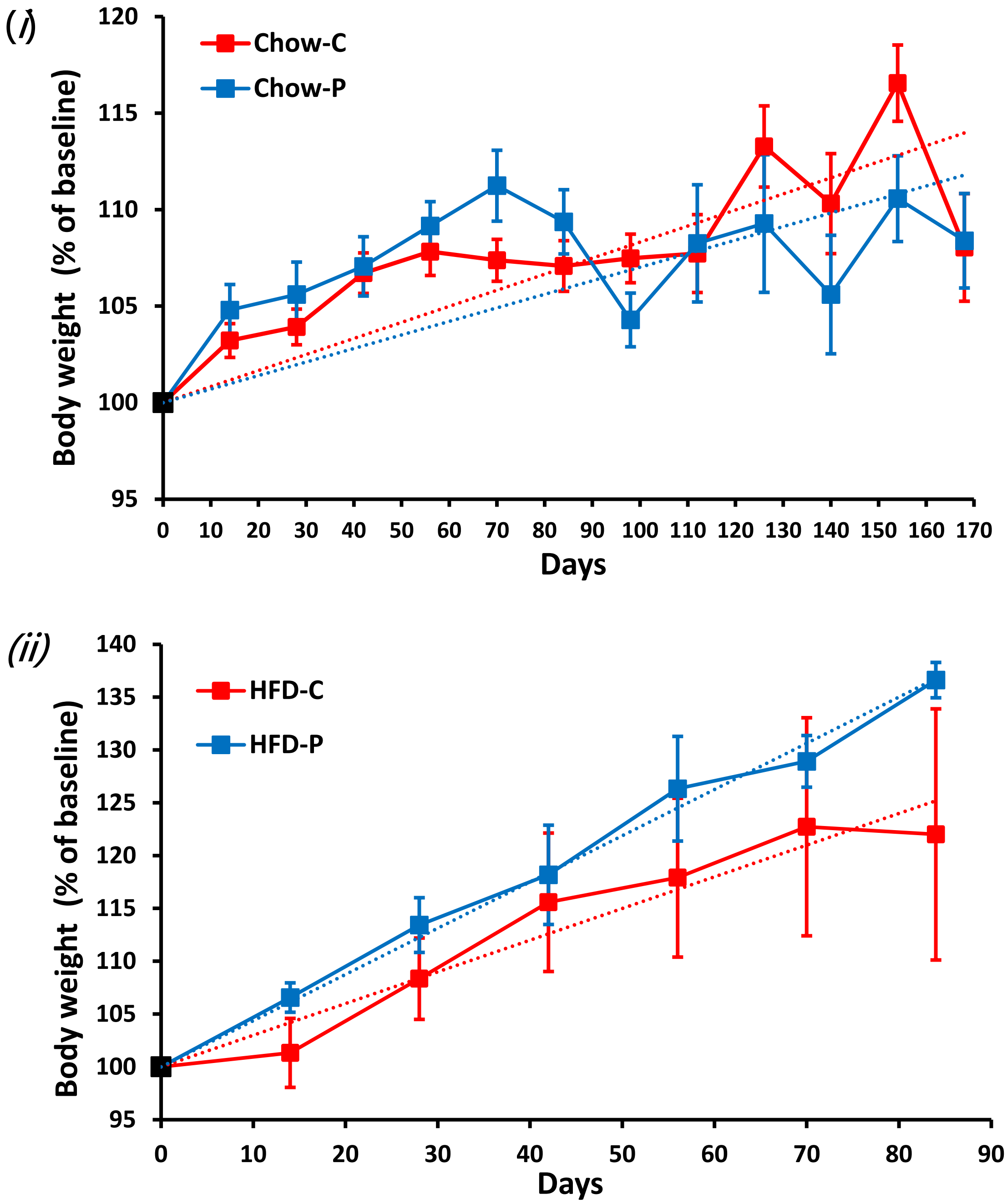

Supplementary Figure S4: Percentage change in body weight in (*i*) 3xTG mice receiving chow alone (Chow-C) or chow with Lab4P (Chow-P) for 24 weeks (Study A) or (*ii*) 3xTg-AD mice receiving HFD alone (HFD-C) or HFD with Lab4P (HFD-P) for 12 weeks (Study B). Data are expressed as mean ± standard error of the mean (SEM) of at least 4 mice per group.

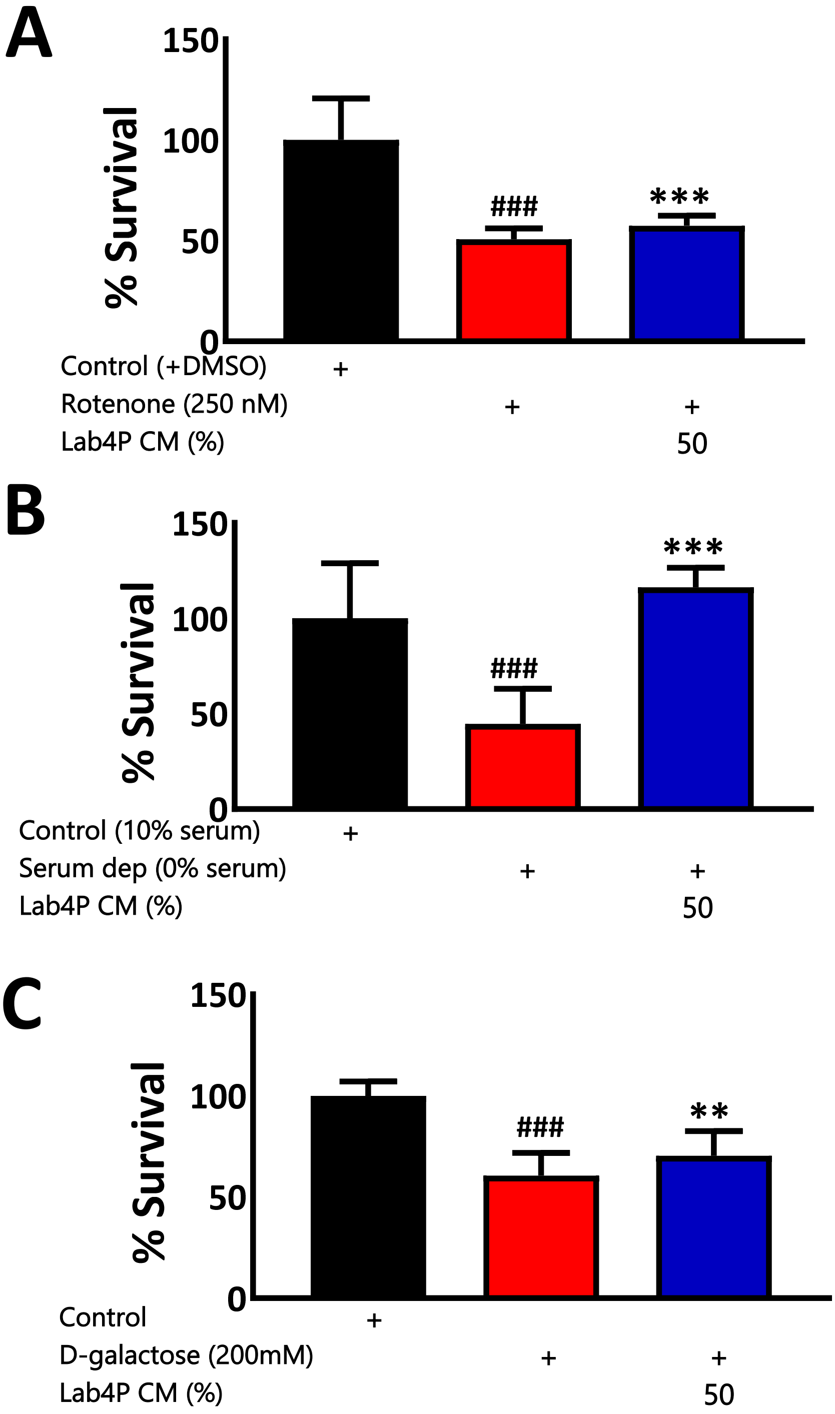

Supplementary Figure S5: The viability of untreated SH-SY5Y cells (undifferentiated, Control) or those treated with (**A**) rotenone (250 nM delivered in DMSO that was included in the control), (**B**) serum deprivation (0% serum) or (**C**) D-galactose (200 mM) with or without 50% Lab4P CM for 48 hours. Viability is expressed as percentage survival compared to the control (black bars) that have been arbitrarily assigned as 100%. The data are presented as the mean ± standard error of the mean (SEM) of at least 3 independent experiments. Values of *p* were determined using one-way ANOVA with Tukey's host hoc analysis where $^{###}p<0.001$ versus the control or $^{**}p<0.01$ or $^{***}p<0.001$ versus the challenged cells (Red bars). DMSO, dimethyl sulphoxide; CM, conditioned media.
